# Protective Effect of α-Linolenic Acid on Non-Alcoholic Hepatic Steatosis and Interleukin-6 and -10 in Wistar Rats

**DOI:** 10.3390/nu12010009

**Published:** 2019-12-18

**Authors:** Camila Jordão Candido, Priscila Silva Figueiredo, Rafael Del Ciampo Silva, Luciane Candeloro Portugal, Jeandre Augusto dos Santos Jaques, Jeeser Alves de Almeida, Bruna de Barros Penteado, Dhébora Albuquerque Dias, Gabriela Marcelino, Arnildo Pott, Rita de Cássia Avellaneda Guimarães, Priscila Aiko Hiane

**Affiliations:** 1 Graduate Program in Health and Development in the Midwest Region, Medical School, Federal University of Mato Grosso do Sul, Campo Grande 79070-900, Brazil; pri.figueiredo92@gmail.com (P.S.F.); jeeser@gmail.com (J.A.d.A.); gabi19ac@gmail.com (G.M.); rita.guimaraes@ufms.br (R.d.C.A.G.); priscila.hiane@ufms.br (P.A.H.); 2 Medical School Clinics Hospital Residency Program, University of São Paulo, USP, Ribeirão Preto 14015-010, Brazil; rafaeldelciampo@hotmail.com; 3 Institute of Biosciences, Federal University of Mato Grosso do Sul, Campo Grande 79070-900, Brazil; lucandeloro@yahoo.com.br; 4 Biochemistry Sector, Institute of Biosciences, Federal University of Mato Grosso do Sul, Campo Grande 79070-900, Brazil; jeandreaugusto@hotmail.com (J.A.d.S.J.); penteadobrunadebarros@gmail.com (B.d.B.P.); dheboradias5@gmail.com (D.A.D.); 5 Research in Exercise and Nutrition in Health and Sports Performance - PENSARE, Graduate Program in Movement Sciences, UFMS, Campo Grande 79079-900, Brazil; 6 Posgraduate Program in Biotechnology and Biodiversity in the Central-West Region of Brazil, Federal University of Mato Grosso do Sul, Campo Grande 79079-900, Brazil; arnildo.pott@gmail.com

**Keywords:** fatty acids, omega-3, inflammation, steatosis

## Abstract

Consumption of omega-3 (n-3) polyunsaturated fatty acids (PUFA) is related to improvement in the inflammatory response associated with decreases in metabolic disorders of obesity, such as low-grade inflammation and hepatic steatosis. Linseed (*Linum usitatissimum*) oil is a primary source of n-3 fatty acids (FAs) of plant origin, particularly α-linolenic acid, and provides an alternative for the ingestion of n-3 PUFA by persons allergic to, or wishing to avoid, animal sources. In our study, we evaluated the effect of the consumption of different lipidic sources on metabolic and inflammatory parameters in Wistar rats. We split 56 male rats into four groups that were fed for 60 days with the following diets: sesame oil, (SO, *Sesamum indicum*), linseed oil (LO), SO + LO (SLO), and a control group (CG) fed with animal fat. Our results reveal that the use of LO or SLO produced improvements in the hepatic tissue, such as lower values of aspartate aminotransferase, liver weight, and hepatic steatosis. LO and SLO reduced the weight of visceral fats, weight gain, and mediated the inflammation through a decrease in interleukin (IL)-6 and increase in IL-10. Though we did not detect any significant differences in the intestine histology and the purinergic system enzymes, the consumption of α-linolenic acid appears to contribute to the inflammatory and hepatic modulation of animals compared with a diet rich in saturated FAs and or unbalanced in n-6/n-3 PUFAs, inferring possible use in treatment of metabolic disorders associated with obesity and cardiovascular diseases.

## 1. Introduction

The impacts of nutrition on health–disease processes are becoming increasingly evident [1]. Changes in eating practices have led to a higher consumption of saturated fatty acids (SFAs) and omega-6 (n-6) polyunsaturated fatty acids (PUFA), as well as a reduction in the ingestion of vegetables, fibers, and omega-3 (n-3) PUFA [2,3,4].

The quality of the consumed fatty acids (FAs) directly influences the adipose tissue, contributing to systemic inflammation through the secretion of pro-inflammatory adipocytokines, such as interleukin (IL)-6 and tumor necrosis factor α (TNF-α), and anti-inflammatory adipocytokines, such as IL-10 [1,5,6]. The state of low-grade inflammation is the pathological characteristic associated with chronical diseases such as obesity, metabolic syndrome (MS), non-alcoholic fatty liver disease (NAFLD), diabetes mellitus type 2 (DM2), and cardiovascular disease (CVD) [7,8,9].

Studies suggest the actuation of IL-6 in inflammation, mainly concerning hypertriglyceridemia associated with visceral obesity, increases insulin resistance and accumulation of hepatic fat, characteristic of non-alcoholic fatty liver disease (NAFLD) or hepatic steatosis [10,11]. IL-10 creates an anti-inflammatory physiological condition in the adipose tissue (AT), inhibiting the production of TNF-α and IL-6, and increasing the levels of the other anti-inflammatory cytokines [12].

The profile of consumed FAs also affects the platelet function and potential stimuli of the formation of thrombi and atheromas. In the formation of atheromatous plaque, the platelets play an essential role, promoting endothelial activation, modulation of the inflammatory phenomenon, and start the formation of lesions and their subsequent thrombotic complications [13].

The FAs of the n-3 series are capable of minimizing the acute and chronic inflammation through various associated effects, whereas the eicosanoids derived from the excess of n-6 PUFAs formed in the organism are potent mediators of thrombosis and inflammation. Therefore, though not quite a consensus, most reports recommend an increase in ingestion of n-3 FA, aiming to lower the proportion of n-6 to n-3 and prevent several pathologies [14]. The mechanisms of the effects of n-3 can include a change in the composition of lipidic membranes, higher energetic expenditure, less formation of reactive oxygen species (ROS), and activation of peroxisome proliferator-activated receptor (PPAR), which act to decrease the activation of inflammatory pathways, such as c-Jun N terminal kinase (JNK) and nuclear factor-κB (NF-κB). Moreover, signaling modular molecules regulate the function of the immune system cells through liberation of cytokines, cell differentiation, and platelet aggregation [9,15,16,17,18].

α-Linolenic acid (ALA) is an n-3 PUFA of plant origin and a food source of eicosapentaenoic acid (EPA) and docosahexaenoic acid (DHA) due to the biochemical conversion of α-linolenic acid (ALA) to EPA, and of EPA to DHA [19]. Though the conversion is around 10% in humans, studies show promising results in the reduction of inflammation and cellular lipotoxicity after the ingestion of ALA. Higher consumption of ALA has been linked to cardiovascular protection, anti-cancer, neuroprotective, and anti-osteoporotic effects, which are critical for evaluating their sources and dosages [20].

Linseed (*Linum usitatissimum* L.) and sesame (*Sesamum indicum* L.) oils are widely consumed and have become the focus of clinical studies due to their functional properties. Their seeds are rich in PUFAs, and a large part of their FAs are present in the form of the essential FAs LA and ALA [14]. Besides containing high concentrations of ALA, linseed oil contains high percentages of bioactive compounds that exert hypolipidemic and antioxidant actions. Its consumption is associated with the reduction in oxidative stress, arteriosclerosis, hypercholesterolemia, and hepatic steatosis [2,7,17,21]. In contrast, sesame oil, besides high quantities of PUFAs, contains lignans of sesamin, sesamolin, and homologues to tocopherol, which are related to improvement in the lipidic profile, reduction of blood glucose, and anti-inflammatory and antiproliferative properties in carcinogenic cells [22,23,24].

In view of the promising results we published on the use of PUFAs of plant origin as a lipid source in Wistar rats evidenced by the positive effects on the lipid and glycemic profile, in this study, our objective was to investigate the effect of linseed and sesame oils, sources of ALA and n-6 PUFA, respectively, on target organs and the inflammatory mediation of the metabolism of Wistar rats.

## 2. Materials and Methods

### 2.1. Raw Material and Diets

Golden linseed and sesame oils were acquired from Pazze Food Industry (Panambi, Rio Grande do Sul, Brazil) and were used to formulate of the experimental diets. We formulated the diets according to the American Institute of Nutrition (AIN), considered as a standard diet for in vivo studies to date for 60 days of treatment [25]. All diets follow the standard recommendation regarding proportions of nutrients, and therefore, are similar in composition, except for the type of oil used, consisting of different lipidic sources: animal fat (lard), sesame oil (SO), linseed oil (LO), and a mixture of SO with LO (an isocaloric diet) [14].

### 2.2. Profile of Fatty Acids and Indices of Nutritional Quality

We based the profiles of fatty acids (FAs) in the diets of the experimental period on a previous analysis (Table 1) [14], which evaluated the total lipidic content using a modification [26] of the method proposed by Hartman and Lago [27]. The FA profile was determined using a gas chromatograph (GC) (Shimadzu GC-2010, Nishinokyo, Japan) with an AOC-5000 auto-injector and flame ionization detector (FID). We used a Restek Stabilwax-DA (Bellefonte, PA, USA) column (fused silica) (30 m × 0.25 mm; 0.25 µm), and both injector and FID operated at 250 °C. The peaks of methyl esters were identified by comparison of their retention times in the column with the retention time of the standards of FA methyl esters, and the quantification was determined according to the American Oil Chemists′ Society (AOCS) Ce 1e-91 method and using an area correction factor [28].

Based on the composition of free FAs, we assessed the nutritional quality using three different indices: the atherogenic index (AI) (Equation (1)), the thrombogenic index (TI) (Equation (2)), [29] and the hypocholesterolemic:hypercholesterolemic (HH) ratio (Equation (3)) [30].

(1) $$\mathrm{AI}\;=\;\frac{C_{12:0}+\;4\;\times {\;C}_{14:0}\;+{\;C}_{16:0}}{\sum^{\text{​}}\mathrm{MUFA}\;+\;\sum^{\text{​}}n6\;+\;\sum^{\text{​}}n3}$$

(2) $$\mathrm{TI}\;=\;\frac{C_{14:0}+{\;C}_{16:0}\;+{\;C}_{18:0}}{0.5\;\times \;\sum^{\text{​}}\mathrm{MUFA}+0.5\;\times \;\sum^{\text{​}}n6+3\;\times \;\sum^{\text{​}}n3+\left(n3/n6\right)}$$

(3) $$\mathrm{HH}\;=\;\frac{C_{18:1\mathrm{cis}9}+{\;C}_{18:2n6}\;+{\;C}_{20:4n6}+{\;C}_{18:3n3}+{\;C}_{20:5n3}+{\;C}_{22:5n3}+{\;C}_{22:6n3}}{C_{14:0}+{\;C}_{16:0}}$$

### 2.3. Animals and Experimental Design

We followed the animal protocols according to the ethical rules and guidelines, and the experimental protocol was approved by the Ethics Committee for Animal Use (protocol no. 681/2015), which is essential in the International Guiding Principles for Biomedical Research Involving Animals (CIOMS), Genebra, 1985; the Universal Declaration of Animal Rights (UNESCO), Bruxelles, Belgium, 1978; and guidelines of the National Health Institutes on the use and care of laboratory animals.

Fifty-six young (21-day-old) Wistar rats (*Rattus norvegicus*) were randomly assigned into four groups, namely: sesame oil (SO), linseed oil (LO), sesame oil + linseed oil (SLO), and a control group with animal fat (CG). The groups were maintained under a 12/12 h light/dark cycle at 22 °C and received one of the four formulated diets for 60 days. The animals were weighed weekly on a semi-analytic balance (Marte-Modelo™ AS 5.500, São Paulo, Brazil) and received food and water ad libitum. The food consumption was weighed (grams/day). At the end of the experiment, after nocturnal fasting, we recorded the body weights. Next, we anaesthetized the rats with isoflurane for blood sampling through the inferior cava vein, and then they were euthanized by bleeding. The visceral fats (epididymal, mesenteric, and retroperitoneal) and the first portion of the intestine and liver were collected and weighed, and the liver and intestine were stored in formalin for later histological analysis.

### 2.4. Serum Markers

We centrifuged, (Fanem^®^, Excelsa II, 206 BL, São Paulo, Brazil) the blood samples to determine the levels of aspartate aminotransferase (AST) and alanine aminotransferase (ALT) using the enzymatic-colorimetric method and spectrophotometry readings [31,32,33].

### 2.5. Assays of Ectonucleotidase Triphosphate Diphosphohydrolase (E-NTPDase) and Ecto-5′-Nucleotidase (E-5′-NT)

We prepared the plasma rich in platelets (PRP) as previously described [34] with modifications [35]. ATP (A6419), ADP (A2754), AMP (A1752), bovine serum albumin (BSA, A2153), trichloroacetic acid (TCA, T4885), 4-(2-hydroxyethyl)piperazine-1-ethanesulfonic acid sodium salt (HEPES-H7006) were acquired from Sigma-Aldrich (St. Louis, MO, USA). All other reagents used in the experiments were of analytic quality and high purity.

We verified the platelet E-NTPDase activity in a reaction medium containing 5 mM CaCl_2_, 100 mM NaCl, 4 mM KCl, 5 mM glucose, and 50 mM Tris-HCl buffer at pH 7.4 and at a final volume of 200 µL [34]. For E-5′-NT, the reaction medium was the same, except that 5 mM CaCl_2_ was replaced by 10 mM MgCl_2_. First, 20 µL platelets (8–12 µg protein) were added to the reaction mixture and pre-incubated at 37 °C for 10 min. The reaction was started by the addition of ATP or ADP to produce a final concentration of 1 mM, and the incubation continued for 60 min. For the hydrolysis of AMP, we assessed the activity of E-5′-NT as described above, and the final AMP concentration was 2 mM. In all cases, the reactions were stopped by the addition of 200 µL TCA at 10% to produce a final concentration of 5%. We determined the liberated inorganic phosphate (Pi) using malachite green/molybdate/polyvinyl alcohol as the colorimetric reagent and KH_2_PO_4_ as the standard [36]. The controls served to correct non-enzymatic hydrolyses of nucleotides. All samples were performed in triplicates. Specific activities of enzymes are reported as nanomole of liberated Pi per minute per milligram of protein. The protein was determined using the Coomassie blue assay [37] with BSA as the standard.

### 2.6. Enzyme-Linked Immunosorbent Assay (ELISA) for Inflammatory Factors

The concentrations of the cytokines IL-6 and IL-10 were determined in the serum of the animals by enzyme-linked immunosorbent assay (ELISA, Peprotech, Rocky Hill, NJ USA) utilizing a specific rat kit, according to the manufacturer’s instructions. The cytokine levels are expressed in pg mL^−1^, and were compared with the standard curve proposed by the specifications of the ELISA kit. 

### 2.7. Histological Analysis

Fragments of liver and intestine collected after euthanasia were fixed in 10% formalin for 12 h and then processed until their inclusion in paraffin. We obtained 5 µm thick sections, stained in hematoxylin and eosin (H&E).

For histopathological analysis of the intestinal mucosa, we observed the intestinal villi and crypts, as well as the presence and intensity of leukocyte infiltration. The intestinal score was classified by the degree of mucosal alteration [38]. The following subdivisions were used according to changes in the intestinal mucosa villi and glands: grade 0, intact mucosa; grade 1, development of Gruenhagen subepithelial space at villus tip; grade 2, presence of cell lysis, Gruenhagen subepithelial space formation, and increased villus spacing; and grade 3, destruction of the free portion of the villi, presence of dilated capillaries, and increase in inflammatory cells.

The morphological analyses of the liver were based on the presence of the following histopathological parameters: steatosis (fatty degeneration), necrosis, vasodilation, leukocyte infiltration, hyaline degeneration, and hydropic degeneration [39]. We used a scoring system: 2, absence of lesion; 4, focal microvesicular steatosis in some liver lobes; 6, diffuse micro and macrovesicular steatosis by the hepatic lobes; and 8, diffuse macrovesicular stenosis by the hepatic lobes. To evaluate the density of hepatic steatosis, we captured 10 images with a 40× objective lens, per liver section (DM 5500 microscope, Leica Microsystems©, Wetzlar, Germany). A screen was used on the computer monitor with a test system containing 36 points [40]. The volume density of hepatic steatosis (Vv [steatosis]) was estimated as the relationship between the points that touched the fat vesicles (Pp) and the number of total points (PT, in this case 36 points) (Equation (4)).

(4) $$Vv\;\left[steatosis\right]\;=\;\frac{\mathrm{PP}\;\left[\mathrm{steatosis}\right]}{PT}$$

### 2.8. Statistical Analyses

Data are expressed as mean and standard deviation (SD). Kolmogorov–Smirnov verified data normality. One-way ANOVA with Bonferroni post hoc was used for parametric data. Histological analyses were not normally distributed and were submitted to the Kruskal–Wallis test with Dunn post hoc. The study power (1-b) was calculated at 0.965. The significance level was set at *p* < 0.05. Microsoft Excel and Graphpad Prism 7.0 were used for statistical analysis

## 3. Results

### 3.1. Profile of Fatty Acids and Indices of Nutritional Quality

The diets were proportional to the FA profile of the fats used (Table 1); CG had the highest SFA content, whereas LO had the highest values of PUFA and predominance of α-linolenic acid (ALA), at 51.89%. SO also showed high levels of PUFAs, but with a predominance of linoleic acid (LA) of 43.58% ± 0.02%, while the mixed diet with both, SLO, presented a balance between PUFA n-6 and PUFA n-3.

The breakdown of FA composition enabled the evaluation of the nutritional quality of the lipid fraction through their indices (Table 2). AI and TI demonstrate the capacity of FAs to promote or prevent atherosclerosis and coronary thrombosis based on their effects on serum cholesterol and concentrations of cholesterol of low-density lipoprotein (LDL). In this study, IA was different amongst all groups: highest in CG, followed by SO, SLO, and LO. The CG had the highest TI, whereas the other diets did not differ amongst them.

The HH considers the effects of FAs on the decrease in metabolic cholesterol; hence, high values are undesirable from a nutritional point of view. The LO diet had the highest HH, followed by SLO, SO, and CG, a reflection of their FA composition.

### 3.2. Ingestion, Weight Gain, Weight of Liver, and Visceral Fats

**Figure 1:**
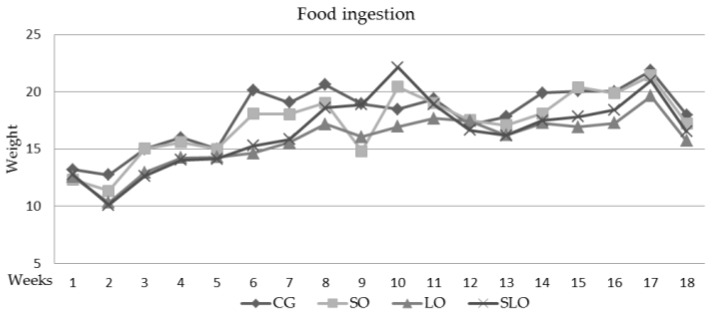
Food ingested in grams (g) by the animals for 60 days of treatment. The groups did not present statistical differences (*p* ≥ 0.05); *n* = 14 rats/group.

Independent of the source of the fat added to the diets, no significant difference was found in the food ingestion by different groups of animals over the 60 days of treatment (Figure 1).

**Figure 2:**
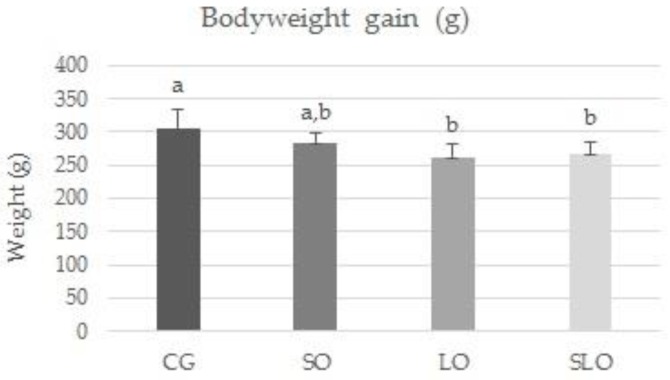
Bodyweight gain of the animas fed diets with different lipid sources over the 60 days of the experiment. Different letters represent statistical differences (*p* < 0.05) by one-way ANOVA followed by post hoc correction Bonferroni test. *n* = 14 rats/group.

Beside the isocaloric diets and the lack of significant differences, the CG animals that consumed more saturated fats gained more weight than the other groups, but this difference was not statistically significant (Figure 2).

Similar results are provided in Table 3 regarding the main visceral fats of the animals (epididymal, mesenteric, and retroperitoneal). The increased PUFA in the diet of the animals, with less SFA, caused less buildup of body fat. SO, LO, and SLO groups had lower visceral fat weights compared with the CG.

The increased n-3 PUFA in the diets was directly correlated with the reduced liver weight (Table 3). The CG and SO groups presented higher weights when compared with LO and SLO groups, which consumed diets containing LO, rich in n-3 ALA.

### 3.3. Biochemical Parameters

Transaminases are essential enzymes that are used for detecting possible hepatic damage. Under normal conditions, they are found inside the hepatocytes but are extravasated due to possible lesions.

The enzymatic activity of aspartate aminotransferase (AST) was, on average, 24% lower in the groups fed diets containing LO and SLO compared with the CG (Table 4). However, the composition in FAs consumed in the diet did not show any alteration in the plasmatic activity of the enzyme ALT amongst the evaluated groups.

### 3.4. E-NTPDase and E-5′-NT Assays

**Figure 3:**
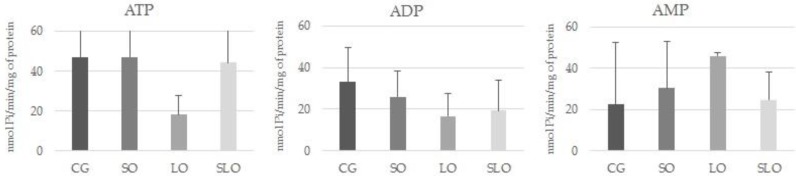
Ectonucleotidase triphosphate diphosphohydrolase (E-NTPDase) and ecto-5′-nucleotidase (E-5′-NT) activity in platelets of rats treated with animal fat, or linseed, sesame, or sesame + linseed. The activity of E-NTPDase was measured using ATP and ADP as substrates. The activity of E-5′-NT was measured using AMP as substrate. The hydrolysis of nucleotides is expressed in nmol Pi/min/mg of protein. Bars represent means ± standard error of the mean (SEM; *p* > 0.05, *n* = 2–8) one-way ANOVA.

To evaluate the functional characteristics of the platelets, we investigated the activity of the enzymes of the purinergic system expressed on the surface of these cells that participate in the regulation of stimulus to the platelet aggregability. E-NTPDase and E-5′-nucleotidase are found anchored in the plasmatic membrane and have their catalytic site oriented to the extracellular medium. Figure 3 shows the activity of E-NTPDase and E-5′-NT in platelets of animals in the groups SO, LO, SLO, and CG, amongst which we observed no significant differences.

### 3.5. IL-6 and IL-10

**Figure 4:**
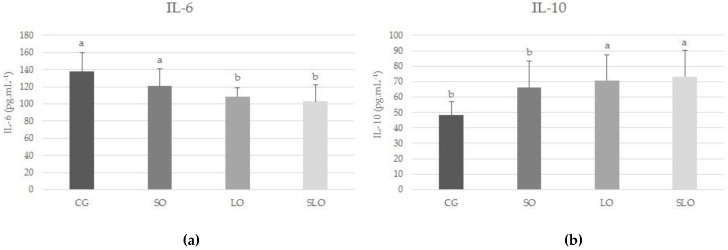
Cytokines related to immunologic and inflammatory conditions in the serum of the animals after 60 days of treatment with diets with different lipidic sources: (**a**) interleukin (IL)-6 and (**b**) IL-10. Statistical differences by one-way ANOVA test and Bonferroni post hoc (*p* < 0.0004 and *p* < 0.02).

To evaluate the effects of various lipidic sources under immunologic and inflammatory conditions, we measured the cytokines involved in the pro-inflammatory process (IL-6) and in the polarization of the macrophages type M1 and M2 (IL-10). The LO and SLO groups showed a significant decrease in IL-6 compared with the control group (*p* = 0.02, Figure 4a) and an increase in IL-10 (*p* = 0.004, Figure 4b). The substitution of saturated fat by PUFA showed a statistically significant difference in improvement of the immunologic and inflammatory conditions.

### 3.6. Histopathology of Liver and Intestine

**Figure 5:**
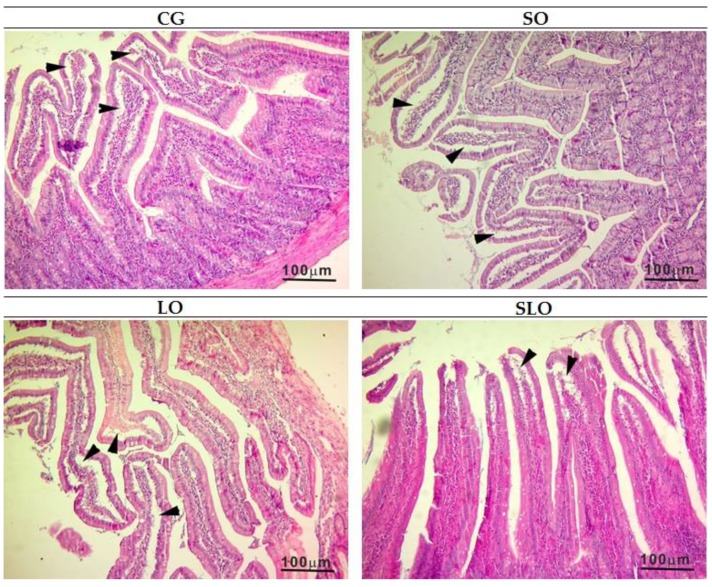
Histological analysis of the intestine of the rats. The arrows indicate the subepithelial space. CG, control group (animal fat); SO, sesame oil; LO, linseed oil; SLO, sesame oil + linseed oil.

The alterations in the intestinal villi were classified using the degree of mucosal alteration [38]. All groups were classified as intestinal villi of grade 1 (light), which is characterized by well-constituted villi, without cellular lysis or inflammatory process, but with the formation of increased spacing among vilosities (Gruenhagen space) (Figure 5).

**Figure 6:**
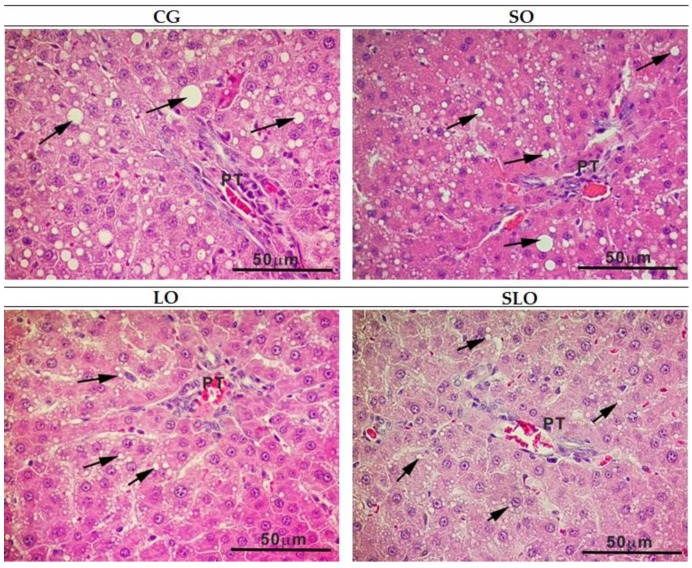
Histological analysis of the liver of the animals in CG, SO, LO, and SLO groups. Arrows indicate hepatic steatosis. CG, control group (animal fat); SO, sesame oil; LO, linseed oil; SLO, sesame oil + linseed oil.

For the evaluation of the density of steatosis, we counted points on laminae stained in H&E. We observed periportal steatosis in all groups located in the peripheric region of the hepatic lobes (zone 1). The steatosis was moderate in the livers of the animals in the CG, SO, and SLO groups, being significantly different compared to the animals in the LO group, in which we observed a light level of steatosis (Table 5). Table 6 shows that liver fat content in CG, SG, and SLO was not statistically different and show differences in relation to the LO group. However, the LO and SLO groups showed a similar statistical standard between them, with the trend in the SLO to be equal to LO and lower than CG and SO groups (Table 5). We also observed that steatosis in groups CG and SO was micro and macrovesicular, periportal, and diffuse throughout the liver, whereas LO and SLO only presented microvesicular steatosis (Figure 6). The difference between SLO and LO groups (Table 6), occurs because the first presented periportal microvesicular steatosis of several lobes, while in the second there was only microvesicular steatosis periportal in some lobes.

## 4. Discussion

The effects caused by the high consumption of SFA and the unbalance in the ingestion ratio between omega-6 and omega-3, arising from the modern and western diet, have influenced the development and evolution of diseases such as obesity, DM2, NAFLD, and their related comorbidities. The ingestion of PUFAs, especially of the n-3 series, is being recommended for their proven benefits to health, mainly regarding prevention, attenuation, and even reversion of these diseases [3,41]. In our study, the decrease of SFA and increase of PUFA in the diet of animals promoted an improvement in the biochemical, inflammatory, and histopathological parameters. The effects were more pronounced when the proportion of n-3 PUFA series was higher than that of the n-6 PUFA series. According to the literature, a ratio of omega-6/omega-3 of 1–2:1 is reported as ideal and one of the dietary factors most important in the prevention of obesity [3].

As AI and TI indicate risk of the formation of thrombi, atheromatous plaques, and plaque aggregation [29], which can lead to the development of cardiovascular diseases, our results showed that the AI and TI of the diets, especially LO (Table 2), were low when compared with other foods in the human diet, such as fish and chicken [42,43]. The data, except for CG, were similar to those reported in evaluations of the hot-pressed linseed and sesame oils, with IA and IT values below 1.0 [44,45].

The hypercholesterolemic index represents the proportion between PUFAs and SFA present in the diet and high values indicate a favorable nutritional balance; thus, the lower the SFA quantities, the lower the tendency to activate inflammatory markers, the risk of cardiovascular diseases, and the accumulation of free FAs in the liver [45,46]. The HH values of 4.82 for SO and 14.85 for LO are superior to those reported for Brazilian fish (1.87–2.18), already considered rather high [47].

At the end of the 60 day experiment, the rats fed with the diets rich in PUFAs exhibited lower accumulated body weight, liver weight, and deposits of visceral fats, only showing alterations in the used lipid sources, without changes in the proportion or caloric value. We highlight that the increase in n-3 PUFA could have influenced the lower body weight gain and liver weight. This is similar to the findings reported for rats supplemented with fish oil, which is the primary source of n-3 FA of animal origin. Those rats presented the lowest weight gain, fat content in carcass, and serum levels of triglycerides (TG), AST, and total cholesterol [48].

As the adipose cells increase in number and size, they start to produce a series of compounds that regulate metabolism, such as peptides and cytokines IL-6 and TNF-α, associated with numerous metabolic disorders [2]. Our study corroborates this evidence, since we observed that the increase in adipose tissue was higher in CG and SO, proportional to increased levels of the pro-inflammatory IL-6 in the animals. In contrast, the LO and SLO groups presented lower levels of circulating IL-6 and higher levels of IL-10, inferring the protection of PUFAs in inflammatory activity.

The capacity of n-3 to reduce IL-6 is linked to lower expression of associated apoptosis protein, transcription factor C/EBP homologous protein (CHOP), and modulation of the expression of XBP1, with the consequent blockage of the activation of the JNK inflammatory pathways and inhibiting nuclear factor-κB (IκB) kinase (IKK) and regulation of the mediators of inflammation [49]. This PUFA can inhibit the production of sterol regulatory element-binding protein (SREBP)-1c, which leads to reduced de novo lipogenesis and accumulation of TG in the liver, resulting in lower organ weight and less liberation of very-low-density lipoprotein (VLDL) and TG in the blood, vital factors for reducing risks related to cardiovascular diseases and metabolic inflammation [18,19,31]. Our results are consistent with the expected action of increased n-3 in the reduction of IL-6 and liver weight in groups LO and SLO, which ingested higher levels of n-3 PUFA (*p* < 0.05) [50]. The increase in IL-10 in the LO group enhanced the protective characteristic of n-3 PUFA since it inhibited the production of IL-6, thereby reducing the pro-inflammatory effects [46,47].

A diet rich in SFA is related to a chronic pro-inflammatory state that directly or indirectly affects platelet function [13,51]. Therefore, to evaluate the functional characteristics of platelets, we investigated the activity of enzymes of the purinergic system that participate in regulation of the stimulus to platelet aggregability, considering the E-NTPDase and E-5′-nucleotidase enzymes, which are involved in the metabolism of the extracellular ATP and its conversion to ADP, AMP, and adenosine [52]. In agreement with the atherogenic and thrombogenic indices (Table 2), the activity of the enzymes in the different investigated groups did not show alterations, indicating a potential stimulus of platelet aggregation and, consequently, the formation of thrombi and atheromas. The length of treatment could have been insufficient to perceive significant alterations in any of the groups.

Besides the inflammatory evaluation, the hepatic damage induced by the diets must be considered as the increase in the circulating AST is a reliable indicator of hepatic lesions and possibly heart attack [31], which was reduced in the groups consuming higher quantities of n-3. Several authors also reported the action of n-3 PUFA in the reduction of the enzymes related to hepatic damage (AST and ALT) [21,53]. Their diminished activity in the LO and SLO groups emphasized the hepatoprotective effects exerted in the animals. Other studies already highlighted the capacity of omega-3 to decrease blood AST and glucose in randomized assays in humans [54] and other models of study in animals [21,53].

The high values of AST in CG and SO groups are suggestive of hepatic damage in these animals, which is one of the clinical signs of NAFLD. NAFLD is related to two main factors: the unbalance in the input and output of FAs, which leads an excessive accumulation of hepatic fat [55], and oxidative stress, which can cause hepatocellular lesions. Our recorded histopathological scores confirm the alterations in AST and the relationship with hepatic damages. The animals in group LO presented a level of steatosis classified as light, whereas the other groups received a classification of moderate (*p* < 0.05). Our results can be attributed to the capacity of n-3 PUFA acting in the positive regulation of PPAR expression, which codifies proteins involved in FA oxidation, and in the negative regulation of SREBP-1 and SREBP-2, codifying the proteins of lipidic synthesis, decreasing the availability of free FAs and hepatic steatosis [56]. Moreover, n-3 PUFA negatively regulates IL-6, with lower stimulation of hepatic TG production. The quantification of points of steatosis indicated a similar lower percentage in the group LO and SLO rats, which had a similar statistical pattern. The CG and SO groups had the highest percentages of steatosis. Hence, we suggest that the ingestion of animal fat and SO causes more accumulation of hepatic fat and that the α-linolenic acid present in LO, isolated or inserted as sources of n-6, such as SO, lessens the formation of steatosis, revealed by histological analysis of livers (Figure 6).

The higher quantity of ALA in LO provided an omega-6:omega-3 ratio of approximately 1:3, whereas the ratio in SLO was close to 1:1, supporting the finding that the lower the proportion of omega-6 to omega-3, the stronger the hepatoprotective effect. Other studies identified hepatoprotection in rats supplemented with n-3 PUFA [21,57]. The mechanisms related to protection exerted by the n-3 FAs include reduction of the activity of acetyl-coenzyme A (CoA) carboxylase and inhibition of de novo lipogenesis in the liver of rats [50], reduced activity of superoxide dismutase and glutathione peroxidase [58], esterification of glycerol [24,53], and reduced synthesis of arachidonic acid by inhibiting the activities of omega-6 desaturases in the linoleic acid of the liver.

Lower values of weight gain, AST, IL-6, and hepatic steatosis were directly correlated with the increase in PUFAs in the diets, and especial n-3 PUFA ALA, which was the major lipid source in the LO diet. Since ALA is the only food source of n-3 PUFA produced from soil and not from the sea, its use can be an alternative to include this FA in the diet of persons who are allergic to meat or who do not consume animal products.

## 5. Conclusions

A diet with higher quantities of omega-3 promoted improvement in all evaluated biochemical, inflammatory, and histopathological parameters. The consumption of linseed oil, rich in n-3 PUFAs, primarily ALA, was found to be beneficial for decreases in body weight gain and visceral fats. Our results demonstrate a direct connection with the lower expression of pro-inflammatory IL-6 and increase of anti-inflammatory IL-10, and lower values of AST with the reduction of hepatic steatosis.

## Figures and Tables

**Table 1:** Profile of experimental diet fatty acids (mg.100 mg^−1^) of control group (CG), linseed (LO), sesame (SO) oils, and SO + LO (SLO).

Fatty Acid (%)	CG	SO	LO	SLO
Capric, C10:0	0.15 ± 0.021	-	-	-
Lauric, C12:0	0.30 ± 0.04	-	0.12 ± 0.01	-
Myristic, C14:0	1.53 ± 0.15	0.24 ± 0.01	0.24 ± 0.01	0.26 ± 0.03
Palmitic, C16:0	23.12 ± 0.96	13.03 ± 0.05	6.20 ± 0.12	9.78 ± 0.50
Palmitoleic, C16:1	1.86 ± 0.09	0.24 ± 0.00	0.11 ± 0.00	0.27 ± 0.02
Heptadecanoic, C17:0	0.38 ± 0.01	-	-	-
Stearic, C18:0	11.32 ± 0.04	3.31 ± 0.03	3.21 ± 0.03	3.44 ± 0.07
Elaidic acid (*trans*), C18:1	0.19 ± 0.04	-	-	-
Oleic, C18:1 (n- 9)	35.53 ± 0.49	32.61 ± 0.08	16.32 ± 0.05	25.92 ± 0.17
Linoleic, C18:2 (n-6)	16.37 ± 0.12	43.89 ± 0.18	15.68 ± 0.01	30.74 ± 0.02
α-Linolenic, C18:3 (n-3)	1.07 ± 0.05	1.08 ± 0.00	51.89 ± 0.14	23.38 ± 0.11
Arachidic, C20:0	0.24 ± 0.01	0.64 ± 0.15	0.10 ± 0.00	0.35 ± 0.02
Cis-11-eicosenic, C20:1	0.80 ± 0.04	0.25 ± 0.01	0.16 ± 0.04	0.21 ± 0.01
Behenic, C22:0	0.14 ± 0.02	0.13 ± 0.00	0.13 ± 0.01	0.22 ± 0.05
Erucic, C22:1	0.27 ± 0.01	-	-	-
SFA	37.18	17.35	10.00	14.04
MUFA	38.65	33.29	16.85	26.40
PUFA	17.44	44.96	67.57	54.12
n-6/n-3 ratio	15.29	40.64	0.30	1.31

SFA—saturated fatty acid; PUFA—polyunsaturated fatty acid; MUFA—monounsaturated fatty acid.

**Table 2:** Indices of nutritional quality calculated from the chromatographic determination of the lipidic fractions of the oils of linseed and sesame and elaborated diets.

Index	CG	SO	LO	SLO
Atherogenic index (AI)	0.53 ± 0.03 ^a^	0.18 ± 0.00 ^b^	0.09 ± 0.00 ^c^	0.013 ± 0.1 ^d^
Thrombogenic index (TI)	1.17 ± 0.04 ^a^	0.40 ± 0.00 ^b^	0.06 ± 0.00 ^b^	0.14 ± 0.01 ^b^
Hypocholesterolemic: hypercholesterolemic (HH)	2.15 ± 0.12 ^d^	5.85 ± 0.03 ^c^	13.03 ± 0.03 ^a^	7.99 ± 0.44 ^b^

Note: Values expressed as mean ± SD. Different letters in the same line, represent statistic difference (*p* < 0.05) by one-way ANOVA followed by Bonferroni test; *n* = 14 rats/group; CG, control group (animal fat); SO, sesame oil; LO, linseed oil; SLO, sesame oil + linseed oil.

**Table 3:** Weight of liver and visceral fats in grams (g) on the euthanasia day of the animals treated for 60 days.

Parameter	CG	SO	LO	SLO
Liver	10.29 ± 1.18 ^a^	10.03 ± 0.67 ^a^	8.68 ± 0.72 ^b^	8.71 ± 0.72 ^b^
Epididymal	8.57 ± 1.91 ^a^	6.24 ± 1.58 ^b^	4.52 ± 1.03 ^b^	4.68 ± 1.24 ^b^
Mesenteric	5.51 ± 1.41 ^a^	4.02 ± 0.94 ^b^	3.55 ± 0.9 ^b^	3.02 ± 0.63 ^b^
Retroperitoneal	8.35 ± 2.08 ^a^	5.96 ± 1.58 ^b^	4.54 ± 0.96 ^b^	4.93 ± 1.78 ^b^

Note: Values expressed as mean ± SD. Different letters in the same line represent statistical differences amongst the groups (*p* < 0.05) by one-way ANOVA followed by Bonferroni test; *n* = 14 rats/group.

**Table 4:** Blood biochemical parameters related to the enzymatic activity of aspartate aminotransferase (AST) and alanine aminotransferase (ALT).

Parameter	CG	SO	LO	SLO
AST (UI/L	117.38 ± 14.0 ^a^	103.00 ± 19.54 ^a^	91.14 ± 9.33 ^b^	90.0 ± 7.93 ^b^
ALT (UI/L)	64.9 ± 12.2 ^a^	73.51 ± 7.4 ^a^	71.0 ± 16.7 ^a^	60.98 ± 10.4 ^a^

Note: Different letters in the same line represent statistic difference amongst groups (*p* < 0.05) by one-way ANOVA followed by Bonferroni test. *n* = 14 rats/group.

**Table 5:** Mean scores of the histopathological evaluations of the liver and intestine of the animals after 60 days of treatment with diets with different lipid sources.

	Score			
**Organ**	**CG**	**SO**	**LO**	**SLO**
Level of liver steatosis	6 ^a^	6 ^a^	4 ^b^	6 ^a^
Evaluation of the intestine vilosities	4 ^a^	4 ^a^	4 ^a^	4 ^a^

Note: Different letters in the same line represent statistic difference amongst groups (*p* < 0.01). Absence of lesion, 2 points; light lesion, 4 points; moderate lesion, 6 points; and intense lesion, 8 points.

**Table 6:** Density of hepatic steatosis amongst groups treated with different lipid sources using counting of points in laminae.

Parameter	CG	SO	LO	SLO
% steatosis	39.91 ± 3.7 ^a^	53.61 ± 1.5 ^a^	18.97 ± 2.10 ^b^	29.72 ± 3.8 ^a,b^

Note: Values expressed as mean ± standard deviation. Different letters in the same line represent statistic difference amongst groups (*p* < 0.05) by one-way ANOVA followed by post hoc Tukey’s test.

## References

[1] Myles I.A. (2014). Fast food fever: Reviewing the impacts of the Western diet on immunity. Nutr. J..

[2] Figueiredo P.S., Inada A.C., Marcelino G., Cardozo C.M.L., Freitas K.C., Guimarães R.C.A., Castro A.P., Nascimento V.A., Hiane P.A. (2017). Fatty Acids Consumption: The Role Metabolic Aspects Involved in Obesity and Its Associated Disorders. Nutrients.

[3] Simopoulos A.P. (2016). An Increase in the Omega-6/Omega-3 Fatty Acid Ratio Increases the Risk for Obesity. Nutrients.

[4] World Health Organization (2014). WHO Regional Office for Europe: European Food and Nutrition Action Plan 2015–2020. www.euro.who.int/__data/assets/pdf_file/0003/294474/European-Food-Nutrition-Action-Plan-20152020-en.pdf?Ua=1.

[5] Fritsche K.L. (2015). The Science of Fatty Acids and Inflammation. Adv. Nutr..

[6] Stolarczyk E. (2017). Adipose tissue inflammation in obesity: A metabolic or immune response?. Curr. Opin. Pharmacol..

[7] Jeyapal S., Kona S.R., Mullapudi S.V., Putcha U.K., Gurumurthy P., Ibrahim A. (2018). Substitution of linoleic acid with α-linolenic acid or long chain n-3 polyunsaturated fatty acid prevents Western diet induced nonalcoholic steatohepatitis. Sci. Rep..

[8] Lucas C., Lucas G., Lucas N., Krzowska-Firych J., Tomasiewicz K. (2018). A systematic review of the present and future of non-alcoholic fatty liver disease. Clin. Exp. Hepatol..

[9] Minihane A.M., Vinoy S., Russell W.R., Baka A., Roche H.M., Tuohy K.M., Teeling J.L., Blaak E.E., Fenech M., Vauzour D. (2015). Low-grade inflammation, diet composition and health: Current research evidence and its translation. Br. J. Nutr..

[10] Calder P.C., Ahluwalia N., Brouns F., Buetler T., Clement K., Cunningham K., Esposito K., Jönsson L.S., Kolb H., Lansink M. (2011). Dietary factors and low-grade inflammation in relation to overweight and obesity. Br. J. Nutr..

[11] Xuguang H., Aofei T., Tao L., Longyan Z., Weijian B., Jiao G. (2019). Hesperidin ameliorates insulin resistance by regulating the IRS1-GLUT2 pathway via TLR4 in hepg2 cells. Phytother. Res..

[12] Medeiros N.I., Mattos R.T., Menezes C.A., Fares R.C.G., Talvani A., Dutra W.O., Rios-Santos F., Correa-Oliveira R., Gomes J.A.S. (2017). IL-10 and TGF-beta unbalanced levels in neutrophils contribute to increase inflammatory cytokine expression in childhood obesity. Eur. J. Nutr..

[13] Gonzalez J., Donoso W., Díaz N., Albornoz M.E., Huilcaman R., Moraes E., Moore-Carrasco R. (2014). High fat diet induces adhesion of platelets to endothelium in two models of dyslipidemia. J. Obes..

[14] Figueiredo P.S., Candido C.J., Jaques J.A., Nunes Â.A., Caires A.R., Michels F.S., Almeida J.A., Filiú W.F., Hiane P.A., Nascimento V.A. (2016). Oxidative stability of sesame and flaxseed oils and their effects on morphometric and biochemical parameters in an animal model. J. Sci. Food Agric..

[15] Calder P.C. (2017). Omega-3 fatty acids and inflammatory processes: From molecules to man. Biochem. Soc. Trans..

[16] Tan C., Voss U., Svensson S., Erlinge D., Olde B. (2013). High glucose and free fatty acids induce beta cell apoptosis via autocrine effects of ADP acting on the P2Y 13 receptor. Purinergic Signal..

[17] Hernández-Rodas M.C., Valenzuela R., Echeverría F., Rincón-Cervera M.Á., Espinosa A., Illesca P., Videla L.A. (2017). Supplementation with Docosahexaenoic Acid and Extra Virgin Olive Oil Prevents Liver Steatosis Induced by a High-Fat Diet in Mice through PPAR-α and Nrf2 Upregulation with Concomitant SREBP-1c and NF-kb Downregulation. Mol. Nutr. Food Res..

[18] Deng X., Dong Q., Bridges D., Raghow R., Park E.A., Elam M.B. (2015). Docosahexaenoic acid inhibits proteolytic processing of sterol regulatory element-binding protein-1c (SREBP-1c) via activation of AMP-activated kinase. Biochim. Biophys. Acta.

[19] Figueiredo P.S., Guimarães R.C.A., Freitas K.C., Hiane P.A. (2018). Physiological and Analytical Approach to Vegetable Oils.

[20] Burdge G.C., Calder P.C. (2005). Conversion of alpha-linolenic acid to longer-chain polyunsaturated fatty acids in human adults. Reprod. Nutr. Dev..

[21] Xu J., Rong S., Gao H., Chen C., Yang W., Deng Q., Huang Q., Xiao L., Huang F. (2017). A Combination of Flaxseed Oil and Astaxanthin Improves Hepatic Lipid Accumulation and Reduces Oxidative Stress in High Fat-Diet Fed Rats. Nutrients.

[22] Moazzami A.A., Haese S.L., Kamal-Eldin A. (2007). Lignan contents in sesame seeds and products. Eur. J. Lipid Sci. Technol..

[23] Aslam F., Iqbal S., Nasir M., Anjum A.A. (2019). White Sesame Seed Oil Mitigates Blood Glucose Level, Reduces Oxidative Stress, and Improves Biomarkers of Hepatic and Renal Function in Participants with Type 2 Diabetes Mellitus. J. Am. Coll. Nutr..

[24] Ismail M., Hasan H., El-Orfali Y., Ismail H., Khawaja G. (2018). Anti-Inflammatory, Antioxidative, and Hepatoprotective Effects of Trans Δ9-Tetrahydrocannabinol/Sesame Oil on Adjuvant-Induced Arthritis in Rats. Evid. Based Complement. Alternat. Med..

[25] Reeves P.G., Nielsen F.H., Fahey G.C. (1993). AIN-93 purified diets for laboratory rodents: Final report of the American Institute of Nutrition ad hoc writing committee on the reformulation of the AIN-76A rodent diet. J. Nutr..

[26] Maia E.L., Rodriguez-Amaya D.B. (1993). Avaliação de um método simples e econômico para a metilação de ácidos graxos com lipídios de diversas espécies de peixes. Rev. Inst. Adolfo Lutz.

[27] Hartman L., Lago R.C.A. (1973). Rapid preparation of fatty acid methyl esters from lipids. Lab. Pract..

[28] Lepage G., Roy C.C. (1986). Direct transesterification of all classes of lipids in a one-step reaction. J. Lipid Res..

[29] Ulbricht T.L.V., Southgate D.A.T. (1991). Coronary heart disease: Seven dietary factors. Lancet.

[30] Santos-Silva J., Bessa R.J.B., Santos-Silva F. (2002). Effect of genotype, feeding system and slaughter weight on the quality of light lambs: Fatty and composition of meat. Livest. Prod. Sci..

[31] Hagen J.H., Hagen P.B. (1962). An enzimatic method for the estimation of glicerol in blood. J. Biochem. Physiol..

[32] Flegg H.M. (1973). An investigation of the determination of serum cholesterol by na enzymatic method. Clin. Biochem..

[33] Carey R.N., Felbruegge C., Westgard J.O. (1974). Evaluation of the adaptation of the glucose oxidase/peroxidase-3-methyl-2-benzothiazoline hydrazone-N, Ndimethylaniline procedure to the technicon SMA 12/60 and comparation with other automed methods for glucose. Clin. Chem..

[34] Lunkes G.I., Lunkes D.S., Morschb V.M., Mazzantib C.M., Morschb A.L.B., Mironb V.R., Schetinger M.R.C. (2004). Ntpdase and 5′-nucleotidase activities in rats with alloxan-induced diabetes. Diabetes Res. Clin. Pract..

[35] Jaques J.A.S., Ruchel J.B., Schlemmer K.B., Pimentel V.C., Bagatini M., do Carmo Gonçalves Souza V., Moretto M.B., Morsch V.M., Schetinger M.R., Leal D.B. (2011). Effects of curcumin on the activities of the enzymes that hydrolyse adenine nucleotides in platelets from cigarette smoke-exposed rats. Cell Biochem. Funct..

[36] Chan K.M., Delfert D., Junger K.D. (1986). A direct colorimetric assay for Ca^2+^-stimulated atpase activity. Anal. Biochem..

[37] Bradford M.M. (1976). A rapid and sensitive method for the quantification of microgram quantities of protein utilizing the principle of protein-dye binding. Anal. Biochem..

[38] Erben U., Loddenkemper C., Doerfel K., Spieckermann S., Haller D., Heimesaat M.M., Zeitz M., Siegmund B., Kühl A.A. (2014). A guide to histomorphological evaluation of intestinal inflammation in mouse models. Int. J. Clin. Exp. Pathol..

[39] Pokorska-S’piewak M., Kowalik-Mikolajewska B., Aniszewska M., Pluta M., Marczynska M. (2017). Novel serum biomarkers modified by the body mass index z-score for the detection of liver fibrosis and steatosis in children with chronic hepatitis C. BMC Infect. Dis..

[40] Aguila M.B., Pinheiro A.R., Parente L.B., Mandarim-de-Lacerda C.A. (2003). Dietary effect of different high-fat diet on rat liver stereology. Liver Int..

[41] Calder P.C. (2014). Very long chain omega-3 (n-3) fatty acids and human health. Eur. J. Lipid Sci. Technol..

[42] Rossano R., Caggiano M.A., Mastrangelo L., Di Lauro R., Ungaro N., Ettorre M., Riccio P. (2005). Proteins, fatty acids and nutritional value in the muscle of the fish speciesmora moro (Risso, 1810). Mol. Nutr. Food Res..

[43] Semwogerere F., Neethling J., Muchenje V., Hoffman L.C. (2019). Meat quality, fatty acid profile, and sensory attributes of spent laying hens fed expeller press canola meal or a conventional diet. Poult. Sci..

[44] Tenyang N., Ponka R., Tiencheu B., Djikeng F.T., Azmeera T., Karuna M.S.L., Prasad R.B.N., Womeni H.M. (2017). Effects of boiling and roasting on proximate composition, lipid oxidation, fatty acid profile and mineral content of two sesame varieties commercialized and consumed in Far-North Region of Cameroon. Food Chem..

[45] Guimarães R.C.A., Macedo M.L.R., Munhoz C.L., Filiu W., Viana L.H., Nozaki V.T., Hiane P.A. (2013). Sesame and flaxseed oil: Nutritional quality and effects on serum lipids and glucose in rats. Food Sci. Technol..

[46] Bersch-Ferreira A.C., Sampaio G.R., Gehringer M.O., Torres E.A.F.S., Ross-Fernandes M.B., da Silva J.T., Torreglosa C.R., Kovacs C., Alves R., Magnoni C.D. (2018). Association between plasma fatty acids and inflammatory markers in patients with and without insulin resistance and in secondary prevention of cardiovascular disease, a cross-sectional study. Nutr. J..

[47] Sousa A.B.B., Junior O.O.S., Visentainer J.V., Almeida N.M. (2017). Total lipid nutritional quality of the adipose tissue from the orbital cavity in Nile tilapia from continental aquaculture. Acta Sci. Anim. Sci..

[48] Devarajan S., Singh R., Chatterjee B., Zhang B., Ali A. (2016). A blend of sesame oil and rice bran oil lowers blood pressure and improves the lipid profile in mild-to-moderate hypertensive patients. J. Clin. Lipidol..

[49] Kandeil M.A., Hashem R.M., Mahmoud M.O., Hetta M.H., Tohamy M.A. (2019). *Zingiber officinale* extract and omega-3 fatty acids ameliorate endoplasmic reticulum stress in a nonalcoholic fatty liver rat model. J. Food Biochem..

[50] Wang X.-H., Li C.-Y., Muhammad I., Zhang X.-Y. (2016). Fatty acid composition in serum correlates with that in the liver and non-alcoholic fatty liver disease activity scores in mice fed a high-fat diet. Environ. Toxicol. Pharmacol..

[51] Monteiro P.F., Morganti R.P., Delbin M.A., Calixto M.C., Lopes-Pires M.E., Marcondes S., Zanesco A., Antunes E. (2012). Platelet hyperaggregability in high-fat fed rats: A role for intraplatelet reactive-oxygen species production. Cardiovasc. Diabetol..

[52] Duan Y., Zeng L., Zheng C., Song B., Li F., Kong X., Xu K. (2018). Inflammatory links between high fat diets and diseases. Front. Immunol..

[53] Ali F.F., Rifaai R.A. (2018). Preventive effect of omega-3 fatty acids in a rat model of stress-induced liver injury. J. Cell. Physiol..

[54] Gonçalves N.B., Bannitz R.F., Silva B.R., Becari D.D., Poloni C., Gomes P.M., Foss M.C., Foss-Freitas M.C. (2018). α-Linolenic acid prevents hepatic steatosis and improves glucose tolerance in mice fed a high-fat diet. Clinics.

[55] Musso G., Gambino R., Cassader M. (2009). Recent insights into hepatic lipid metabolism in non-alcoholic fatty liver disease (NAFLD). Prog. Lipid Res..

[56] Clarke S.D. (2001). Polyunsaturated fatty acid regulation of gene transcription: A molecular mechanism to improve the metabolic syndrome. J. Nutr..

[57] Mustonen A.M., Kärjä V., Kilpiö M., Tammi R., Tammi M., Rouvinen-Watt K., Halonen T., Nieminen P. (2013). Manifestations of fasting-induced fatty liver and rapid recovery from steatosis in voles fed lard or flaxseed oil lipids. Nutrients.

[58] Soleimani A., Taghizadeh M., Bahmani F., Badroj N., Asemi Z. (2017). Metabolic response to omega-3 fatty acid supplementation in patients with diabetic nephropathy: A randomized, double-blind, placebo-controlled trial. Clin. Nutr..

